# Fiber counts and architecture of the human dorsal penile nerve

**DOI:** 10.1038/s41598-023-35030-w

**Published:** 2023-05-31

**Authors:** Elçin Tunçkol, Leopold Purkart, Lennart Eigen, Imre Vida, Michael Brecht

**Affiliations:** 1 Bernstein Center for Computational Neuroscience Berlin, Humboldt-Universität zu Berlin, Philippstrasse 13, Haus 6, Berlin, Germany; 2 Institute for Integrative Neuroanatomy, Charité - Universitätsmedizin Berlin, CCM, Philippstrasse 12, 10115 Berlin, Germany; 3 NeuroCure Cluster of Excellence, Humboldt-Universität zu Berlin, Berlin, Germany

**Keywords:** Nervous system, Peripheral nervous system

## Abstract

The human penis transmits behaviorally important sensory information via the dorsal penile nerve, which is required for initiation and maintenance of erection. The human penis differs from the penes of other hominids. The lack of a baculum makes the human penis dependent on erectile tissue, which is under control of neural signals activated by tactile stimulation. Accordingly, the penile sensory innervation is crucial for human sexual behavior. To clarify penile innervation, we analyzed the architecture of the dorsal penile nerve of five male subjects who donated their body. We stained the sensory fibers in the penile dorsal nerve with anti-neurofilament H antibody, and identified myelinated axons with Luxol fast blue staining. Furthermore, we visualized nerve bundles as they travel along the shaft of the penis by performing microfocus computed tomography scans after counterstaining penes with iodine. Our results show that the dorsal penile nerve is organized in 25–45 loosely packed nerve bundles, running mediodorsally in the shaft of the penis. This organization corresponds to that in penes of other mammalian species, but differs from the organization of the other peripheral sensory nerves. Around half of the dorsal penile nerve fibers were myelinated and a human hemipenis contained a total of 8290 ± 2553 (mean ± SD) axons. Thus, the number of sensory axons in the human dorsal penile nerve is higher than in other species described so far. The large fraction of unmyelinated nerve fibers suggests that the conduction speed is not a crucial aspect of penile sensory transmission.

## Introduction

The human penis is a unique organ in size, structure and function. It is noticeably longer than the penis of other hominids^1^, and unlike all other simians and many other mammals, it lacks a baculum^2^. The lack of a penis bone provides the human penis with enhanced flexibility, but results in a strong reliance on the erectile penile tissue. In response to relevant stimuli, the penis is engorged by increased blood flow and decreased venous drainage of sinuses of erectile bodies, namely the corpora cavernosa and corpus spongiosum. These erectile bodies preserve the erection until the sensory stimulation is ceased or the sexual climax (ejaculation) is reached^3^.

The evolution of the human penis has a peculiar pattern. Comparative studies of penis length^4^ and morphology^5^ have explored primate penis evolution. One of the most acknowledged hypotheses is that the extraordinary penis length of humans might be explained by female choice^1^ and sperm competition^6^. In particular, researchers have indicated the greater penis length and testicle size^7^ in primate species, in which females mate with multiple males, as evidence for such an evolutionary scenario. Others, however, have questioned the evidence for a relation between penis length and sperm competition^8^.

Compared to the general interest in the human penis and the rich behavioral biology work on this topic, the neuroscience of the human penis seems underdeveloped. In particular, we lack a comprehensive understanding of the sensory functions of the human penis. Early studies showed that penile sensation is critical to successful erection and intromissionin primates^9,10^ with subsequent rodent data confirming this conclusion^11^. However, we still lack the data to compare the penile sensitivity of humans and other primates. Given this dearth of data, we set out to answer basic questions about the sensory innervation of the human penis.

Specifically, we addressed the following questions: (i) Where is the sensory-information-transmitting human dorsal penile nerve located? (ii) How are the nerve bundles of the human dorsal penile nerve organized? (iii) What is the pattern of myelinization of dorsal penile nerve fibers? (iv) How many dorsal penile nerve fibers run in a human penis?

## Materials and methods

### Penile tissue preparation

Formalin-fixed penis samples were collected from 5 males who participated in the body donation program of the Center for Anatomy, Charité—Universitätsmedizin Berlin. The donors gave written consent for the use of their bodies for educational and research purposes before they deceased. All the related documents are stored in the department for body donation of the Center of Anatomy. Since this study does not include living human subjects, according to German law no further ethical approval was required. All procedures were carried out in accordance with the “Law for the regulation of the anatomical dissection”(Sektionsgesetz, SRegG BE)^12^ and the “Law on body and burial services” (Bestattungsgesetz, BestattG BE) of the federal state of Berlin, Germany^13^. Personal information of donors was confidential and unknown to the researchers except for their age. The age range of donors was 67–91 and the mean age was 83.4. After further dissection, samples were preserved in 70% alcohol for two days. Subsequently, the tissue was embedded in paraffin as previously described^14^. Paraffin blocks were cut into 10 μm sections on a Leica Jung RM2035 rotary microtome (Leica Instruments GmbH, Germany, Cat# 042819658) and mounted on Thermo Scientific Superfrost Ultra Plus® Gold slides. Consecutive slides were prepared for immunohistochemistry, Luxol fast blue and Nissl staining.

### Immunohistochemical staining

Prior to antibody staining, sections were deparaffinized and rehydrated in a descending ethanol series^28^. For immunohistochemical staining of penile tissue, we performed heat-induced epitope retrieval (HIER) using a citrate buffer (Antigen Unmasking Solution, Citric Acid Based, pH 6, 100× concentrated stock solution, Vector Laboratories Cat# H-3301, RRID: AB_2336227). We used IHC-Tek™ Epitope Retrieval Steamer Set (IHC WORLD, Cat# IW- 1102). The slides were placed in Coplin jars filled with citrate buffer and steamed for 45 min. Afterwards, slides were left to cool for 30 min at room temperature. Antibody staining was performed according to standard procedures. Penis sections were pre-incubated for an hour at room temperature in a blocking solution (0.1 M PBS, 2.5% Bovine Serum Albumin and 0.5% Triton X-100). We prepared nerve fiber stains with primary antibodies against Neurofilament H (Chicken polyclonal, Millipore Cat# AB5539, RRID: AB_11212161) diluted (1:1000) in 0.5% Triton X-100 and 1% Bovine Serum Albumin in 0.1 M PBS; and sections were incubated for at least 48 h at 4 °C. Subsequently, sections were incubated with secondary antibodies, coupled to the fluorophore Alexa 488 (Thermo Fisher Scientific Cat# A-11039, RRID: AB_2534096). The secondary antibodies were diluted (1:1000) in 1% Bovine Serum Albumin in 0.1 M PBS and the reaction was allowed to proceed overnight in the dark at 4 °C. Finally, the sections were mounted on glass slides with Fluoromount G® mounting medium (Biozol, Eching, Germany, Cat# Nr.: SBA-0100-35) and cover slipped.

### Luxol fast blue staining

In order to visualize myelinated fibers, we used the Luxol fast blue stain. After deparaffinization, slides were treated with 1% solvent Blue 38 (Sigma, Cat# S3382) dissolved in 96% ethanol and acetic acid at 56˚C overnight. The slides were rinsed and differentiated using lithium carbonate (American MasterTech Scientific, Cat# KC2622) and 70% ethanol. The tissue was cleared with 100% ethanol and Xylol (Supplementary Table S1). Sections were mounted with Eukitt® (Sigma-Aldrich, Cat#03989).

### Nissl staining

After deparaffinization, sections were dehydrated in a descending ethanol series. After treatment with Cresyl violet, sections were cleared with Xylol and Mounted using Eukitt® (Sigma-Aldrich, Cat#03989).

### Microscopy

Z-stack images were obtained using a Leica DM5500B epifluorescence microscope with a 63x oil objective (axial resolution 0.772 μm) with 1 μm axial steps. The images obtained were from 1600 × 1200 to 8696 × 7706 pixels in size with a field of view between 203 × 152 μm and 1103 × 977 μm. Image stacks were analyzed using the Fiji version of ImageJ (RRID: SCR_002285).

### MicroCT imaging

We used diffusible iodine-based contrast enhanced computed tomography (diceCT) to analyze and visualize penile structures and nerves. To enhance the contrast for microCT a penile section in 1.5 cm thickness was incubated in 1% iodine solution for 1 week. Another entire penis was incubated in 1% iodine solution for 2 weeks. Computed tomography scans were obtained with a YXLON FF20 CT system (YXLON International GmbH, Hamburg, Germany; RRID: SCR_020903). Scans were performed with an isotropic voxel size of 10.6 µm. Images were visualized and segmented using an extended version of the Amira software (AmiraZIBEdition 2022, Zuse Institute Berlin, Germany). Segmentation was done manually with a combination of the ‘Threshold’, ‘Brush’ and ‘Lasso’ module.

### Statistical analysis

We used SPSS Statistics version 29 (IBM, New York, USA; RRID: SCR_002865) for statistical analysis. The normality of the data was tested with one sample Kolmogorov-Smirnov test and the properties of distributions were described.

### Ethical approval

All procedures performed in this study were carried out in accordance with German and international law. Human samples were collected from 5 males who participated in the body donation program of the Center for Anatomy, Charité—Universitätsmedizin Berlin. The donors gave written consent for the use of their bodies for educational and research purposes before they deceased. All procedures were carried out in accordance with the “Law for the regulation of the anatomical dissection” (Sektionsgesetz, SRegG BE) and the “Law on body and burial services” (Bestattungsgesetz, BestattG BE) of the federal state of Berlin, Germany. Personal information of donors was confidential and unknown to the researchers except for their age.

## Results

### Composition of dorsal penile nerve

**Figure 1:**
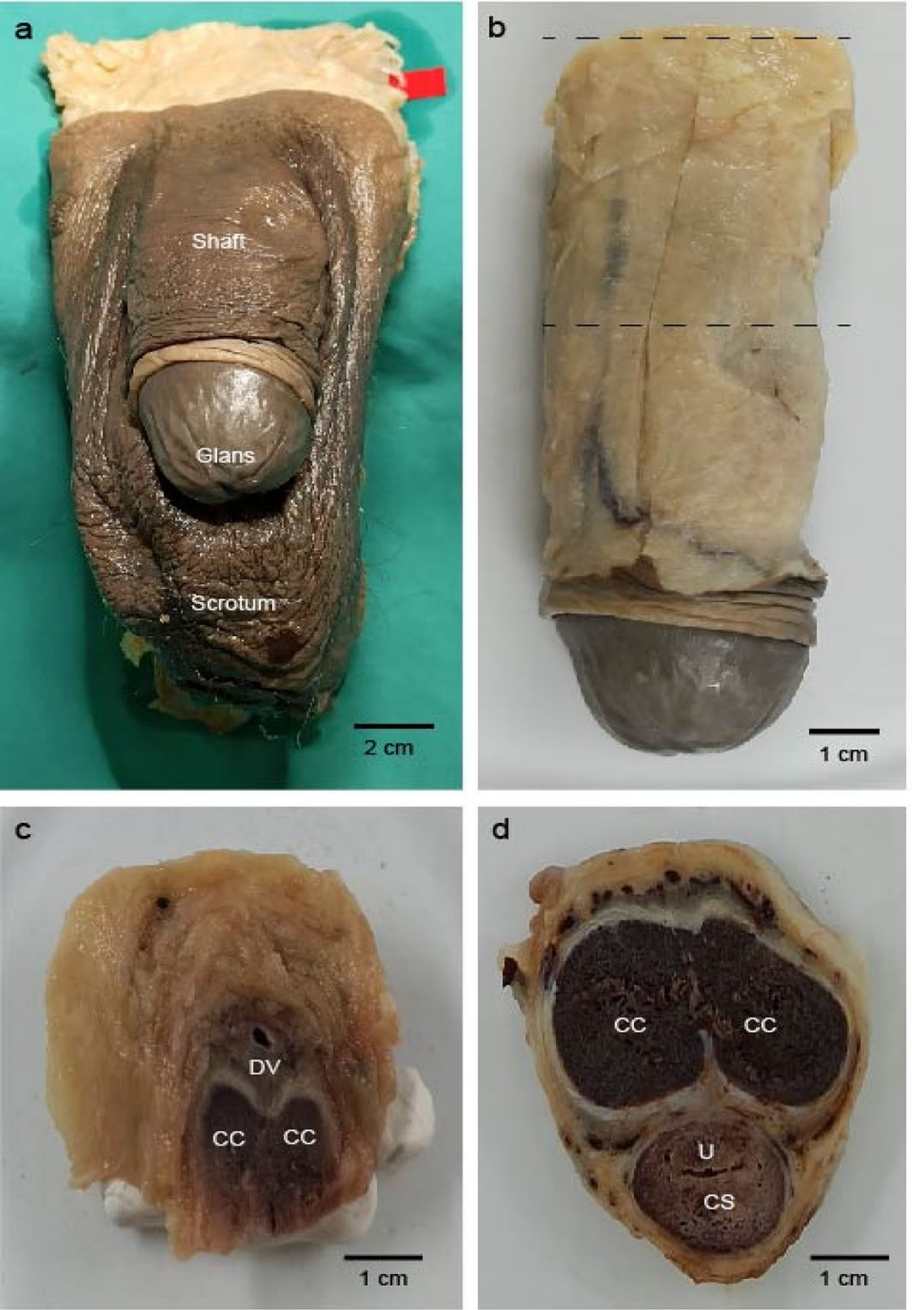
External structure, gross anatomy and sectioning of the human penis. (**a**) External structure of the male genitalia in a frontal view. (**b**) The skinned penis detached from pubic symphysis. Note the buckled structure of the flaccid penis. Dashed lines show where we took the samples shown in (**c**) (upper line) and (**d**) (lower line). (**c**) Cross section of the proximal shaft of the penis shows superficial dorsal vein (DV) surrounded by connective tissue positioned above two corpora cavernosa (CC). The samples were collected from where the penis is attached to the body as shown with the dashed line in panel (**b**). (**d**) Cross section of the distal shaft of the penis shows erectile bodies, corpus spongiosum (CS) and urethra (U). The samples were collected from towards the middle of the shaft as shown with the dashed line in panel (**b**).

In order to investigate the composition of the dorsal penile nerve, we analyzed samples from 5 male subjects from the body donation program of the Charité. First, the skin and the subcutaneous connective tissue of the penis shaft were removed (Fig. 1A). Samples for histology were taken from the base of the shaft below the symphysis (Fig. 1B). In the proximal shaft, the deep dorsal vein of penis was positioned mediodorsally to two corpora cavernosa. The dorsal penile nerve bundles were localized laterally and ventro-laterally to the dorsal vein in the fascia of the penis. (Fig. 1C). Distally, dorsal nerve bundles branched out and continued to spread laterally in the fascia (Fig. 1D).

### MicroCT scanning and segmentation of dorsal penile nerve

**Figure 2:**
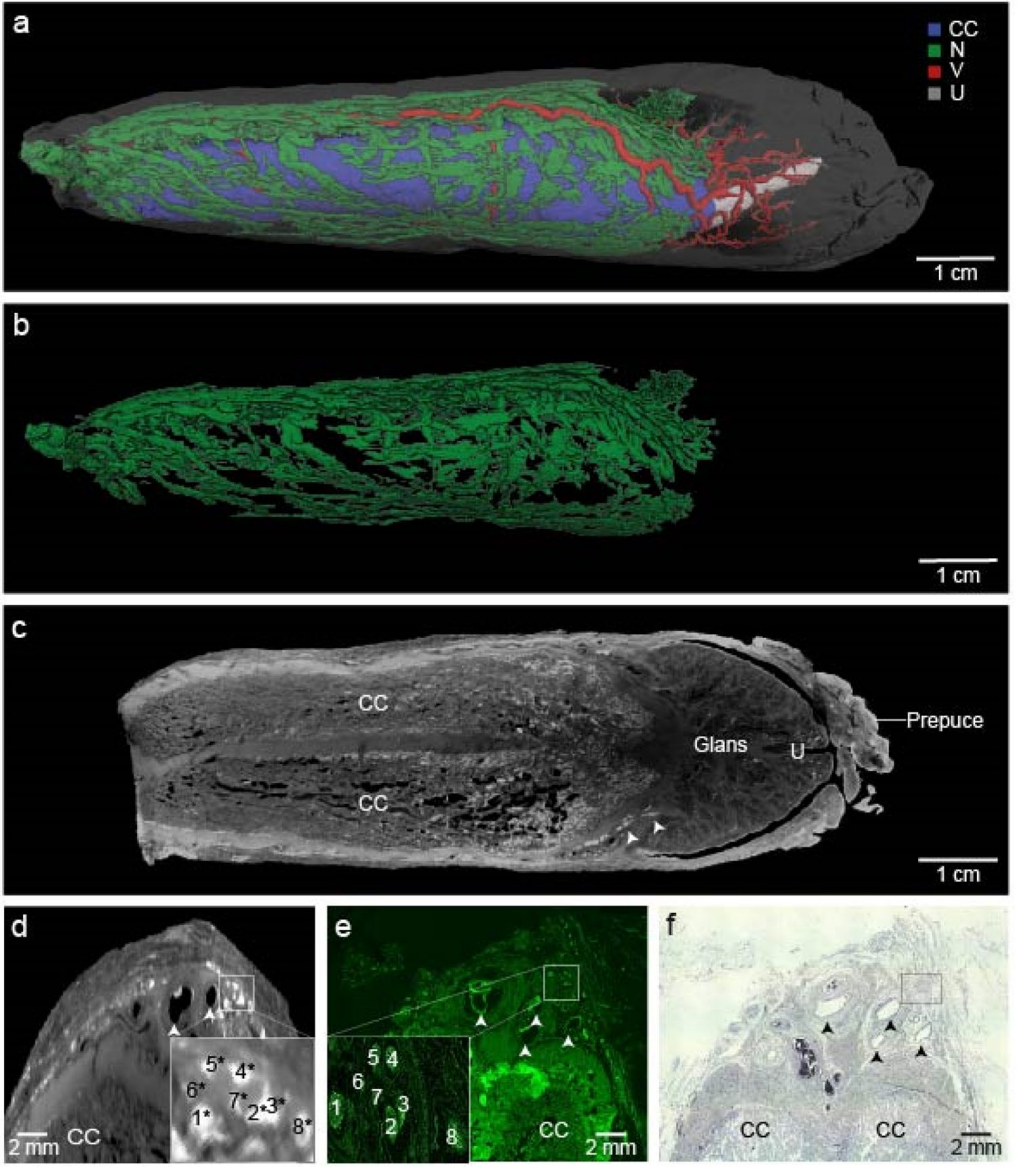
MicroCT scan of the human penis and nerve segmentation. (**a**) Lateral view of microCT scan and segmentation of the penis. Corpora cavernosa (CC), blood vessels (V), urethra (U) and nerve bundles (N) are represented in different colors. (**b**) Volume rendering of segmented nerve branches in the penis. (**c**) Transverse plane of microCT scan showing the anatomical structure of the penis. Arrows show dorsal nerve branches entering to glans penis. (**d**) Coronal section displaying the proximal shaft of the penis. Arrows show the blood vessels. Some bundles are highlighted and numbered. Asterisks indicate where we estimated the position of bundles present in panel (**e**). (**e**) More proximal sections of the same penis were stained against NF-H (green fluorescence). Coronal section shows the same structures and same bundles in panel (**d**). (**f**) Nissl-stained coronal section of the proximal shaft of the same penis.

Another penis was treated with 1% iodine and scanned with a microCT scanner. Nerve bundles and other penile structures were segmented manually (Fig. 2A). Nerve bundles branched out profusely as they traveled distally (Fig. 2B), and entered into the glans penis from dorsomedial and dorsolateral aspects of corpora cavernosa (Fig. 2C). Blood vessels followed a similar fashion of branching as nerve bundles; in fact, they were found in close proximity to nerve bundles (Fig. 2D–F).

The most proximal part of the penis was prepared for paraffin embedding and stained with anti-neurofilament H in 10 µm sections (Fig. 2E). Some antibody-positive bundles are highlighted and numbered (Fig. 2E inset). To check the accuracy of segmenting we matched the NF-H positive bundles with microCT images. Since antibody staining and microCT slices were not consecutive, we utilized the position of veins and relative positions of bundles to identify them. In this penis, we observed 8 bundles (asterisks in Fig. 2D inset) with a good match between nerve bundles putatively identified in the microCT scan and nerve bundles identified by antibody staining. To further confirm the positions of bundles, Nissl staining was performed (Fig. 2F).

### Myelination of penile dorsal nerve

**Figure 3:**
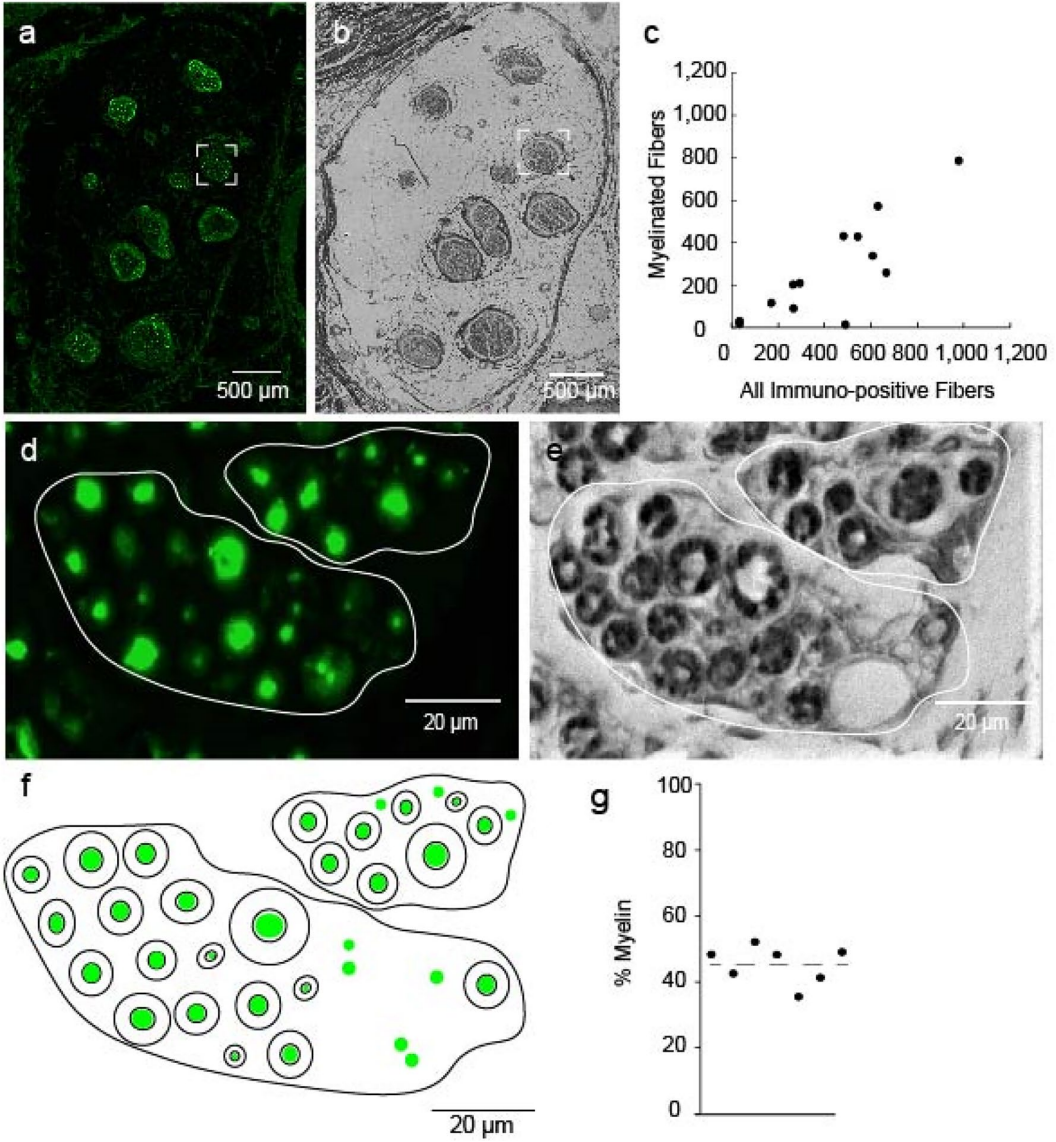
Myelinization of axons in the human dorsal penile nerve. (**a**) Nerve bundles of hemipenis stained with anti-neurofilament H antibody (green fluorescence), a pan-neuronal stain for peripheral sensory axons. One nerve bundle is marked by a dashed square. (**b**) Same bundles in an adjacent 10-µm section were stained with Luxol fast blue to reveal myelin. The dashed square marks the same bundle as in (**a**). (**c**) Plot of counts of myelinated fibers against counts of all neurofilament H-positive fibers (i.e. counts, which putatively include all axons) for the nerve bundles shown in (**a**) and (**b**); each dot corresponds to one nerve bundle, respectively. (**d**) High magnification view of a region of the bundle in the highlighted panel (**a**). (**e**) Same area as in D in an adjacent section stained with Luxol fast blue to reveal myelin. (**f**) Schematic of myelinated and unmyelinated fibers identified from the micrographs shown in (**d**) and (**e**). Green marks represent neurofilament H-positive fibers. Black doughnuts represent myelin sheaths stained with Luxol fast blue. Green marks without black circles represent unmyelinated fibers. Note the smaller diameter of unmyelinated fibers. (**g**) Plot of the fraction of myelinated fibers in the proximal shaft of 7 hemipenes.

In order to characterize the composition of dorsal penile nerve fibers, we combined immunostaining for neurofilament H (Fig. 3A) with Luxol fast blue staining for myelin (Fig. 3B) in consecutive sections. Nerve bundles of the dorsal penile nerve fibers showed a clear myelin staining, further confirming that our antibody staining identified nerve bundles. When the total number of myelinated fibers was plotted against all immuno-positive fibers for each individual bundle (n = 14) (Fig. 3C), we observed that entries were falling below the unity line indicating that only a fraction of penile nerve fibers is myelinated. Moreover, the fraction of myelination varied substantially from bundle to bundle, suggesting that nerve bundles of the dorsal penile nerve carry functionally heterogenous information. The distribution of myelination fraction of individual bundles was moderately skewed in negative direction. High magnification pictures are provided for a subregion of the bundle highlighted in Fig. 3A and B (Fig. 3D and E). In their schematic overlay, green dots represent neurofilament H-positive fibers, while black double-circles represent myelin sheaths (Fig. 3F). Note the small diameter of unmyelinated nerve fibers. We calculated the fraction of myelinated fibers in all immune positive fibers for 7 hemipenes (Fig. 3G). We found that on average, 45% of fibers in the proximal shaft of penes are myelinated. The analysis of myelination suggests that nerve bundles of the dorsal penile nerve are functionally heterogeneous and that a large fraction of penile nerve fibers consists of small unmyelinated—putatively slow conducting—axons.

### Quantification of dorsal penile nerve fibers

**Figure 4:**
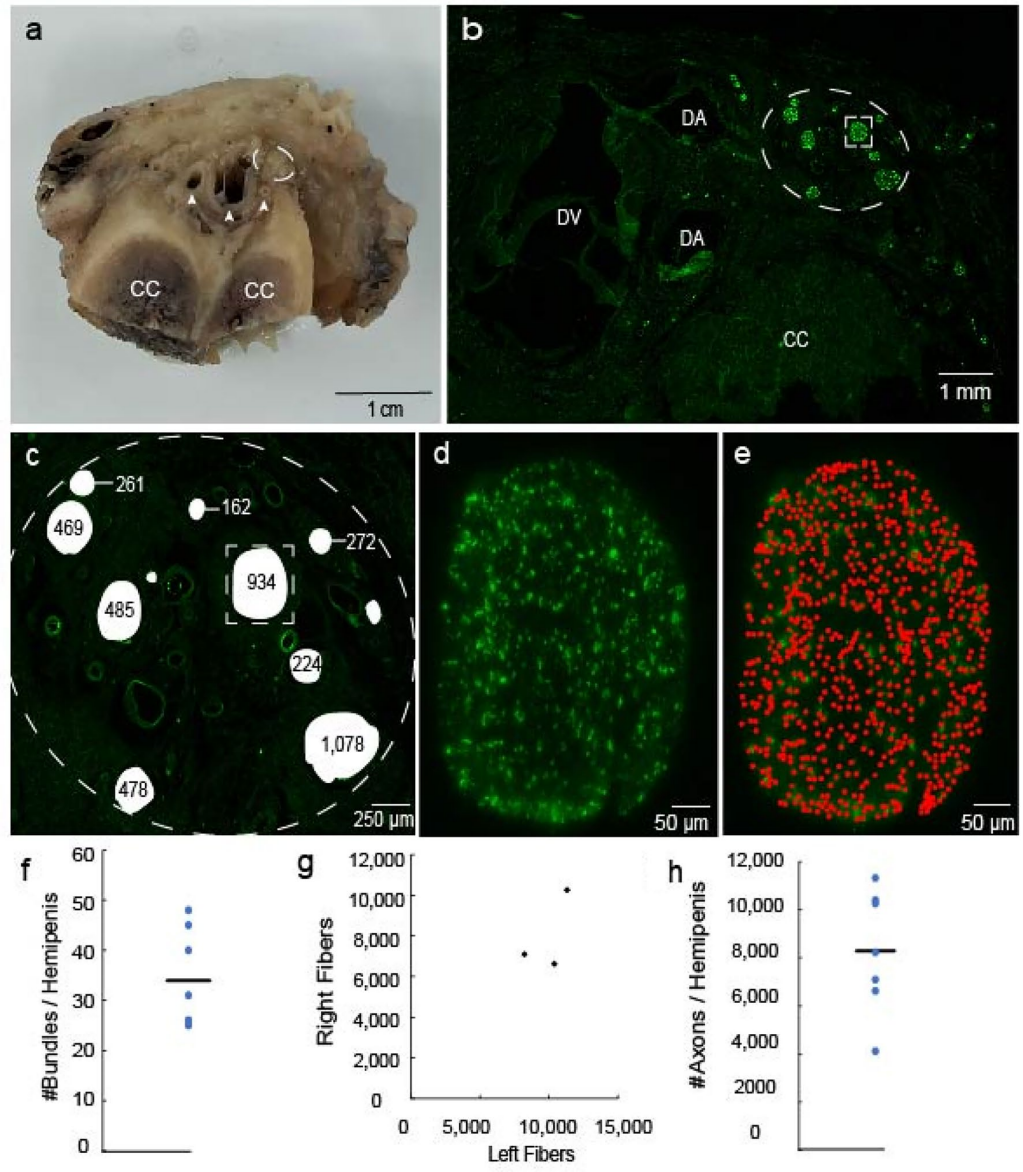
Nerve fiber counts of human dorsal penile nerve. (**a**) Excised section of the human penis analyzed in panel a-e. Arrows indicate arteries and dorsal vein. (**b**) Dorsal nerve bundles identified by neurofilament H-positive staining (green fluorescence) are shown. Bundles are located close to dorsal vein (DV) and dorsal arteries (DS) dorsal to corpus cavernosum (CC). (**c**) Region outlined (dashed) in panel B is shown in higher magnification. Results from axon counts (neurofilament H-positive fibers) are superimposed. Thicker bundles contain more fibers. (**d**) High magnification micrograph of the bundle is shown in the dashed square in (**c**). (**e**) A red dot is superimposed on each fiber counted in the bundle. (**f**) Plot of the number of bundles for 7 hemipenes. The line shows the mean number of bundles. (**g**) Plot of the relationship between the total number of fibers in left and right sides for each individual. (**h**) Plot of the total number of axons in each hemipenis. The line shows the mean axon count.

In order to demonstrate how we quantified the dorsal penile nerve fibers another penis specimen is displayed (Fig. 4A). A section of this specimen was stained with anti-neurofilament H antibodies, and several example nerve bundles were encircled (Fig. 4B). The number of neurofilament H-positive fibers is given for each of the bundles in the circled area (Fig. 4C). A high magnification micrograph of the bundle highlighted (framed by the square in panel B) is shown together with a dot display of our count (Fig. 4D and E). Our data from proximal shaft of 7 hemipenes showed that the dorsal nerve consisted of 35 ± 10 (Mean ± SD) bundles on average for a hemipenis (Fig. 4F). A one sample Kolmogorov-Smirnov test showed that the number of bundles was normally distributed, D(7) = 0.24, *p* = 0.05. We also plotted the relationship between the counts of the left and right sides of individual penes (Fig. 4G) and did not observe any marked divergence. Lastly, we counted the total numbers of fibers for each hemipenis (Fig. 4H). On average there are 8290 ± 2553 (Mean ± SD) fibers in a hemipenis. The total number of fibers count was found to be normally distributed, D(7) = 0.21, *p* = 0.05. We conclude that the human penis contains large number of axons in the dorsal penile nerve.

### Dorsal nerve fibers in the root of the penis

In four other samples we quantified the nerve fibers of the penis root. In the root, nerve bundles are located in close proximity to each other and encapsulated by connective tissue. In 8 hemipenes we analyzed, there are 16 ± 3 (Mean ± SD) big bundles on average with a normal distribution, D(8) = 0.23, *p* = 0.05. Those bundles contained a total of 4389 ± 1661(Mean ± SD) nerve fibers. A one-sample Kolmogorov-Smirnov test showed that the distribution was not normal (D(8) = 0.35, *p* > 0.05), but had a slightly skewed, platykurtosis shape. Finally, we found that 60% of these fibers are myelinated (See Supplementary Fig. S2 online).

## Discussion

We studied the organization and fiber composition of the human dorsal penile nerve in five male subjects. We visualized the penile organization by microCT scans and antibody staining of serial sections. By visualizing nerve fibers, we found that in a hemipenis ~ 8300 dorsal penile fibers run in 25–45 spatially separated bundles, about half of which are myelinated.

The dorsal penile nerve is one of the three main terminal branches of the pudendal nerve and enters the penis bilaterally from root of the penis, running mediodorsally onto the shaft^15^. It bifurcates into lateral and dorsal branches that innervate mainly the shaft and the glans, respectively^16^. In fact, the microCT-based tracing of dorsal penile nerve fibers enabled the visualization of dorsal penile nerve branching in great detail. The nerve carries somatosensory information from free nerve endings as well as corpuscle-like complex receptors^15,17,18^. These inputs constitute essential sensory information for initiating cascade of events leading to erection and ejaculation. Besides autonomic centers in the spinal cord, tactile information is also relayed to subcortical^19^ and cortical targets^20–22^.

We found that on average 45% of dorsal penile nerve fibers were myelinated. This finding is roughly in line with the findings of Purkart and colleagues^14^ that 57% of mouse dorsal penile nerve fibers are myelinated. Therefore we can conclude that most of the fibers innervating the human penis are unmyelinated. However, in the root of the penis more than half of the fibers are myelinated. Previous research showed that dorsal nerve ramifies substantially as it travels down to the glans penis; and the main branches decrease in diameter^16^. In glans penis ramifications perforate into spongy tissue and innervate the skin^23^. Unmyelinated and thin myelinated fibers generate free nerve endings, whereas large myelinated fibers innervate corpuscle-like receptors^17,24^. Therefore, the decrease in myelination of fibers in proximal shaft compared to the root is consistent with the general pattern of ramification of dorsal nerve, which can be also observed in our microCT scan.

Unmyelinated tactile fibers are crucial in the processing of gentle touch, temperature and pain^25^. Gentle stroking of hairy skin activates the unmyelinated C-tactile fibers and incites a feeling of pleasantness in humans^26,27^. Further studies show that unmyelinated fibers project to the posterior insula in a somatotopic fashion^28–30^. Even though there is no consensus on the existence of C-tactile fibers in human genitalia, some studies show gentle stroking of inner thighs incites erotic sensations^31^. Tactile stimulation of the penis in an erotic context leads to activation of the posterior insula^32^. Similar results were obtained when men were presented erotic material and their insula^33^, which receives visceral, somatosensory and nociceptive signals, was activated. Another fMRI study showed that slow-speed gentle stimulation of penile shaft did not cause significant activity in posterior insula but the same stimulation to frenulum deactivated the default-mode networks significantly. This suggests that genital skin may contain small diameter unmyelinated C-tactile fibers; but erotic sensations and sexual feelings may rely on a more complex sensory processing system^34^.

Previous studies investigated the different fibers in the glans penis and in the dorsal penile nerve. Glans penis is found to contain almost only somatic fibers; whereas both somatic and visceromotor (autonomic) fibers are present in the dorsal penile nerve at the level of the root^35^. However, to our knowledge, no quantification of the number of fibers has been carried out in humans. In general, few studies focus on the quantification of the nerve in non-human mammalian species. The first such study quantified the osmium tetroxide stained myelinated fibers in the dorsal penile nerve of rats and found 1423 myelinated fibers on average^36^. A similar study was conducted in rhesus monkeys and found 2170 fibers, unilaterally^9^. In a recent study, immunostainings with the protein gene product 9.5 (PGP 9.5) and neurofilament H were used to visualize all fibers in the mouse dorsal penile nerve and counted 1572 fibers on average^14^. Our study thus shows that the number of nerve fibers in the human dorsal penile nerve is substantially higher than in other mammals, plausibly reflecting the larger physical size as well as the high functional relevance of sensory information for human sexual behavior. However, similar studies with other hominids might reveal the relationship between disproportionate elongation of human penis and the number of fibers in dorsal penile nerve.

The sexual behavior of humans is exceptional^37^, and sensory information is highly important for successful reproduction^9,10^. Since humans lack a baculum, penile rigidity depends on erectile tissue controlled by neural signals, which are activated by tactile stimulation. Previous studies show that the thickening of tunica albuginea on the dorsal penis contributes to the formation of the distal ligament, which acts like a trunk of glans penis. It protects the glans penis and the distal urethra from the pressure generated during coitus^38^ fulfilling the functions of a baculum^39^. Another hypothesis is that human glans penis evolved symmetrical lobes compared to other primates to facilitate erection and rigidity^2^. Therefore, the disappearance of baculum might have allowed symmetrical glans lobes to evolve while creating the necessary space for dorsal thickening to form the distal ligament in the center of corpus spongiosum.

Challenges in the study of penile innervation are the variety of the methods that are used and the limitations that are imposed by the use of human tissue. Thus, it is difficult to assess the effects of aging. Another drawback is our lack of understanding the myelination process and functional differentiation of sensory fibers. In our samples we have observed that in some bundles almost half of the fibers were unmyelinated and segregated from myelinated ones topographically. In order to understand the behavioral importance of myelination in the dorsal penile nerve, further work is required. One of the factors that play a role in glial differentiation is Neuregulin 1(NRG1). Especially NRG1 type III is thought to regulate the degree of myelination in sensory and motor axons depending on threshold levels. Axons expressing more NRG1 III are myelinated more by Schwann cells than those expressing less NRG1 III^40^. Comprehension of the myelination pattern and how the dorsal penile nerve functions are also important for the regenerative and surgical applications^41^. Finally, we note that as a result of the restricted availability of human material our study is limited to a relatively small sample size. We conclude that the comparative and quantitative work on penile innervation can help understanding human sexual organs.

## Supplementary Information

Supplementary Information.

## Data Availability

The data in the manuscript will be made available upon request to the corresponding author.
