# Supplementary material for: Fiber counts and architecture of the human dorsal penile nerve
Source: Sci Rep. 2023 May 31;13:8862. doi: 10.1038/s41598-023-35030-w (PMC10232416; doi:10.1038/s41598-023-35030-w)
Supplement: Supplementary file 1 — Supplementary Information. [file 41598_2023_35030_MOESM1_ESM.pdf]

Fiber Counts and Architecture of the Human Dorsal Penile Nerve

Elçin Tunçkol[(1)], Leopold Purkart[(1)],Lennart Eigen[(1)], Imre Vida[(2)] & Michael Brecht[(1,3,*)]

[(1)] Bernstein Center for Computational Neuroscience Berlin, Humboldt-Universität zu Berlin, Philippstrasse 13, Haus 6, Berlin, Germany

[(2)] Institute for Integrative Neuroanatomy, Charité - Universitätsmedizin Berlin, Philippstrasse 12, CCM, 10115 Berlin, Germany

[(3)] NeuroCure Cluster of Excellence, Humboldt-Universität zu Berlin, Berlin, Germany

[(*)] To whom correspondence should be addressed. Email: michael.brecht@bccn-berlin.de

Supplementary Table S1. Luxol fast blue protocol to stain myelinated fibers

Step	Reagent	Duration
1	Xylol	5 min
2	Xylol	5 min
3	100 % ethanol	3 min
4	100 % ethanol	3 min
5	96 % ethanol	1 min
6	Luxol fast blue	Overnight
7	96 % ethanol	Few seconds
8	Distilled water	Few seconds
9	Lithium carbonate	30 sec
10	70 % ethanol	30 sec
11	Distilled water	Few seconds
12	100 % ethanol	3 min
13	100 % ethanol	3 min
14	Xylol	5 min
15	Xylol	5 min

Supplementary Figure S2. Bundle and axon count in the dorsal penile nerve at the root of penis

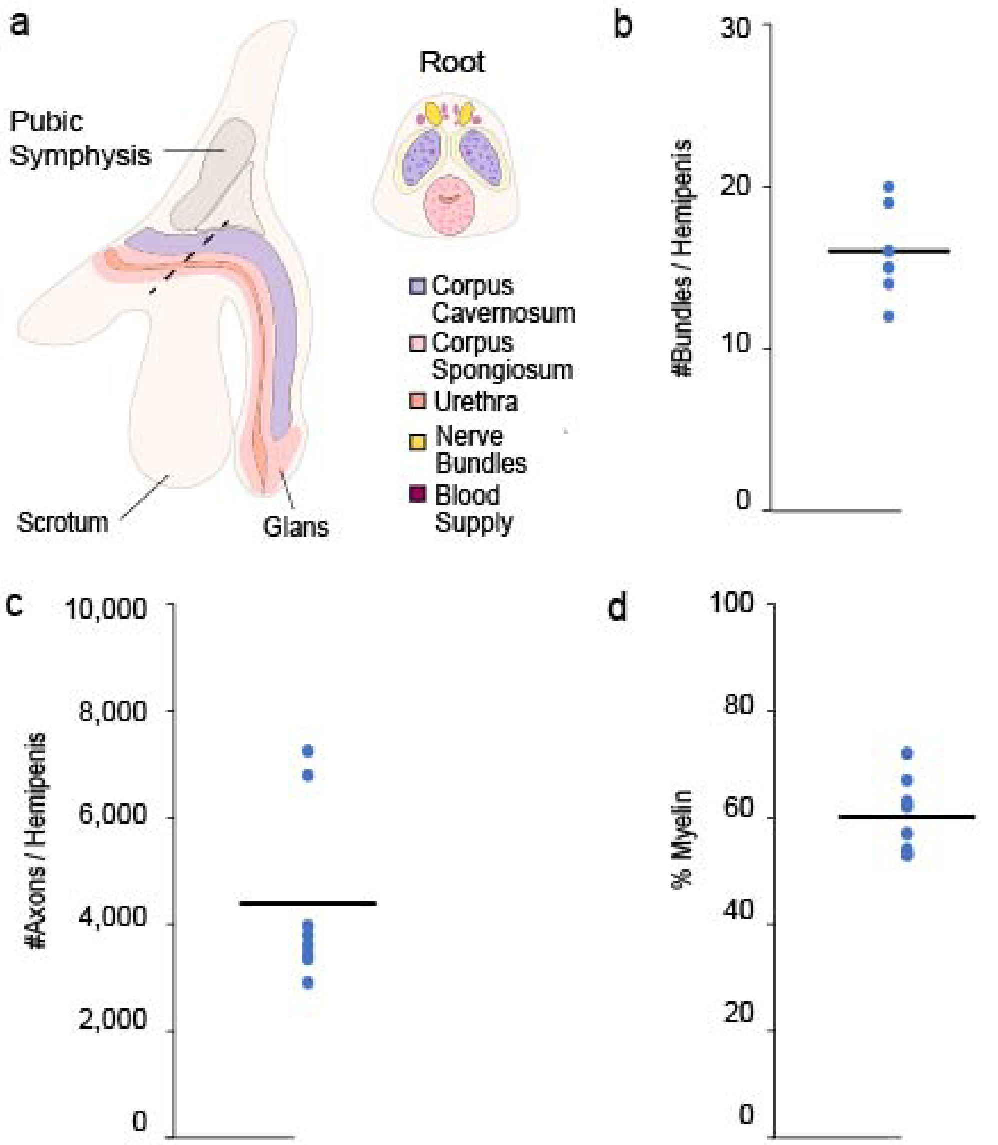

(a) Dashed line shows where we took root samples from. On the right-hand side the penile structures and nerve bundles are depicted.
(b) Plot of the number of bundles for 8 hemi-penes. The line shows the mean number of bundles.
(c) Plot of the total number of axons in each hemi-penis. The line shows the mean axon count.
(d) Plot of the fraction of myelinated fibers in the proximal shaft of 8 hemi-penes.
